# Characterization of Source-Specific Air Pollution Exposure for a Large Population-Based Swiss Cohort (SAPALDIA)

**DOI:** 10.1289/ehp.10177

**Published:** 2007-08-14

**Authors:** L.-J. Sally Liu, Ivan Curjuric, Dirk Keidel, Jürg Heldstab, Nino Künzli, Lucy Bayer-Oglesby, Ursula Ackermann-Liebrich, Christian Schindler, team the SAPALDIA

**Affiliations:** 1 Institute of Social and Preventive Medicine, University of Basel, Basel, Switzerland; 2 Department of Environmental and Occupational Health Sciences, University of Washington, Seattle, Washington, USA; 3 Infras, Zürich, Switzerland; 4 ICREA (Institució Catalana de Recerca i Estudis Avançats) and Center for Research in Environmental Epidemiology (CREAL) at Institut Municipal d’Investigació Mèdica (IMIM), Barcelona, Spain

**Keywords:** cohort study, cumulative exposure, dispersion model, exposure assessment, long-term exposure

## Abstract

**Background:**

Although the dispersion model approach has been used in some epidemiologic studies to examine health effects of traffic-specific air pollution, no study has evaluated the model predictions vigorously.

**Methods:**

We evaluated total and traffic-specific particulate matter < 10 and < 2.5 μm in aero-dynamic diameter (PM_10_, PM_2.5_), nitrogren dioxide, and nitrogen oxide concentrations predicted by Gaussian dispersion models against fixed-site measurements at different locations, including traffic-impacted, urban-background, and alpine settings between and across cities. The model predictions were then used to estimate individual subjects’ historical and cumulative exposures with a temporal trend model.

**Results:**

Modeled PM_10_ and NO_2_ predicted at least 55% and 72% of the variability of the measured PM_10_ and NO_2_, respectively. Traffic-specific pollution estimates correlated with the NO_x_ measurements (*R*^2^ ≥0.77) for background sites but not for traffic sites. Regional background PM_10_ accounted for most PM_10_ mass in all cities. Whereas traffic PM_10_ accounted for < 20% of the total PM_10_, it varied significantly within cities. The modeling error for PM_10_ was similar within and between cities. Traffic NO_x_ accounted for the majority of NO_x_ mass in urban areas, whereas background NO_x_ accounted for the majority of NO_x_ in rural areas. The within-city NO_2_ modeling error was larger than that between cities.

**Conclusions:**

The dispersion model predicted well the total PM_10_, NO_x_, and NO_2_ and traffic-specific pollution at background sites. However, the model underpredicted traffic NO_x_ and NO_2_ at traffic sites and needs refinement to reflect local conditions. The dispersion model predictions for PM_10_ are suitable for examining individual exposures and health effects within and between cities.

Long-term exposure to air pollution, especially particulate matter (PM), has been linked to reduced lung capacity (Ackermann-Liebrich et al. 1997; Gauderman et al. 2004), elevated mortality (Dockery et al. 1993; Filleul et al. 2005; Jerrett et al. 2005; Krewski et al. 2005; Künzli et al. 2000; Lipfert et al. 2006), lung cancer (Nyberg et al. 2000; Vineis et al. 2006), and cardiopulmonary mortality (Gehring et al. 2006; Pope et al. 2002; Rosenlund et al. 2006). Except for the Stockholm, Sweden, studies (Nyberg et al. 2000; Rosenlund et al. 2006), exposure assessment in most air pollution epidemiologic studies generally have used central-site measurements to represent community-wide cohort exposure. This has attracted critiques on inadequate characterization of the long-term exposure of study subjects. The availability of the PM measurements, the types of PM monitors deployed, differences in PM sources, and various degrees of spatial variability in PM could also result in different types of bias in allocating exposure to subjects in separate cities.

To minimize exposure misclassification and in some cases to focus on traffic-related air pollution, recent cohort studies improved previous exposure assessment methodologies by assigning individual exposure indices, including subjective traffic assessment (Heinrich et al. 2005), distance between a major road and residences (Bayer-Oglesby et al. 2006; Garshick et al. 2003; Venn et al. 2001; Vineis et al. 2006), and traffic density/counts near residences (Nicolai et al. 2003; Zmirou et al. 2004). Individual and/or residential outdoor nitrogen dioxide measurements from a subset of cohort were also used for health assessment (Schindler et al. 1998; Sunyer et al. 2006). Other studies constructed statistical models by regressing home outdoor nitrogen oxides or NO_2_ measurements against traffic characteristics (Carr et al. 2002) or local geographic characteristics related to traffic (Brauer et al. 2006; Hochadel et al. 2006; Hoek et al. 2002) for estimating individual residential outdoor concentrations. These statistical models provide improvements over the earlier qualitative indices. However, they used short-term measurements (usually 1- to 2-week averages in 2–4 seasons) to attain annual averages in a specific year. Models constructed from such measurements implicitly assumed that temporal variation is homogeneous within a given area. In addition, the spatial pattern was assumed to hold over the years when such models were applied to long-term exposure estimation.

The dispersion modeling approach is an alternative for assigning individual exposure indices based on both physical and stochastic processes. It is seldom used because detailed emission and meteorologic data are required. Although the dispersion modeling approach has been applied to the Stockholm and Oslo, Norway, cohorts (Bellander et al. 2001; Nafstad et al. 2003, 2004; Nyberg et al. 2000; Pierse et al. 2006; Rosenlund et al. 2006), model evaluation has been limited to comparisons of the modeled NO_2_ against measurements at six monitoring sites (Nyberg et al. 2000). Cyrys et al. (2005) compared dispersion and stochastic model estimates using NO_2_ and PM_2.5_ (particulate matter with aero-dynamic diameter < 2.5 μm) measurements at 40 sites in Munich, Germany, and reported that NO_2_ dispersion predictions overestimated the measured values but were highly correlated with stochastic model estimates. However, the dispersion model used by Cyrys et al. was run without source-specific emission data and predicted total suspended particulate (TSP) which could not be validated directly against PM_2.5_ measurements.

We used a dispersion model to estimate individual exposure to source-specific PM_10_ (particulate matter with aerodynamic diameter < 10 μm), NO_x_, and NO_2_ for subjects in the Swiss Cohort Study on Air Pollution and Lung Diseases in Adults (SAPALDIA) (Ackermann-Liebrich et al. 2005). In this article we provide a detailed evaluation of the dispersion model predictions against fixed-site measurements between and across cities and estimate individual historical exposure to source-specific PM_10_.

## Methods

### Monitoring sites

The SAPALDIA cohort included 9,651 subjects in Switzerland, with the first health examination (SAPALDIA 1) conducted in 1991 (Martin et al. 1997) and a follow-up assessment (SAPALDIA 2) of 8,047 subjects in 2002 (Ackermann-Liebrich et al. 2005). The study areas included two large cities (Basel, Geneva), two medium-sized cities (Aarau, Lugano), two rural areas (Payerne, Wald), and two alpine areas (Davos, Montana). Each area was monitored with up to three fixed monitoring sites for PM, NO_2_, and NO_x_. For dispersion model evaluation, we used measurements from all available Swiss sites, including up to 57 PM_10_ sites, 103 NO_2_ sites, and 17 NO_x_ sites [Supplemental Table 1 (online at http://www.ehponline.org/docs/2007/10177/suppl.pdf)].

### Fixed-site PM measurements

At most central sties, 24-hr PM was measured using the Digitel monitor (High Volume Sampler DHA80; Digitel Elektronik, Hegnau, Switzerland) for TSP between 1990 and 1996 and for PM_10_ afterward. At Davos an FAG β-attenuation monitor was used for TSP and the Harvard impactor (Marple et al. 1987) for PM_10_ (Monn et al. 1995), and a custom-made PM_10_ sampler was used in Aarau between 1990 and 1998. Since 1999, all sites have used the Digitel monitor for PM_10_. A conversion factor of 0.86 was used to estimate PM_10_ from TSP measurements based on a collocation study (Gehrig and Hofer 1999).

### Black smoke measurements

In 2000–2002, black smoke (BS) from PM_2.5_, a marker for traffic exhaust (Brunekreef et al. 1997; Zhu et al. 2002), was measured at nine sites using the Digitel monitor with glass fiber filters. Filters were analyzed gravimetrically and for light reflectance using a Smoke Stain reflectometer (M43D EEL; Diffusion Systems Ltd., London, UK). The absorption coefficient (m^−1^) was calculated using ISO (International Standard) 9835 (Götschi et al. 2002).

### NO_2_ and NO_x_ measurements

NO_2_ and NO_x_ were measured with the Monitor Labs 8840 (Environment SA, Englewood, CO, USA), the Tecan CLD 502 (Tecan, Hombrechtikon, Switzerland), and the Horiba monitor (model APNA-350E; Horiba Europe, Leichlingen, Germany). In 2003, NO_2_ was also measured outside residences with the passive Palmes tubes (Monn et al. 1998; Palmes et al. 1976), integrated over three 2-week periods from 335 residences (Hazenkampvon Arx et al. 2004). These home outdoor measurements in 2003 were used to calibrate the within- and between-city variations from the dispersion model that used emission data in 2000.

### Dispersion modeling

We modeled annual average concentrations of PM_10_, PM_2.5_, NO_x_, and NO_2_ in 1990 and 2000 using the PolluMap Gaussian dispersion model (version 2.0) [Swiss Agency for the Environment, Forests, and Landscape (SAEFL) 2003, 2004]. PolluMap used transfer functions to represent the impact of a source to the neighboring areas. The dispersion was performed for each emission inventory, including road, rail, and air transports, industrial and commercial, construction, household (heating), and agricultural activities, and forestry. For 1990 and 2000, we computed traffic emissions for the road network for passenger cars, light-duty vehicles, motorcycles, and buses (SAEFL 1997). Emissions from heavy-duty vehicles were computed separately from an updated road network and a new relative distribution of traffic loads. We obtained emissions from other sources from the Swiss inventory data (SAEFL 2003, 2004). The emission strengths were allowed to vary by season and hour of the day for stationary sources, and with the day of the week and the hour of the day for transport sources. We computed road transport emissions for all major roads individually and then projected onto the 200 ×200-m grids. For all other source categories, the total emission load was estimated and then spatially disaggregated by distributing it equally to all grid cells with certain land use characteristics. We used different transfer functions for three Swiss regions (alpine, plateau, and others) and for different source types (area vs. line) and heights (0–2, 2–20, and > 20 m). The dispersion modeling was performed for up to 5 km for NO_x_ and 200 km for PM. Dispersion of primary particles was modeled with hourly emission and meteorologic parameters and outputs were averaged for the year.

We calculated secondary inorganic particles, including nitrate, sulfate, and ammonium, by applying a transformation function (European Union, Directorates-General XI 1997) to the smoothed annual average concentrations of NO_2_, NH_3_, and SO_2_. We computed secondary organic matter from 32 classes of anthropogenic and biogenic volatile organic compounds, each from a detailed emission inventory (Bundesamtes für Bildung und Wissenschaft 1995; SAEFL 1995), multiplied by a fractional aerosol yield coefficient and dispersed by the Gaussian model. The concentrations of the secondary particles were averaged over a 12-km radius area around the source to account for the transition time from gaseous precursor to secondary particles.

We added a background concentration to all computed primary and secondary PM_10_ concentrations originating from Swiss sources in the emission inventory data, which captured most emissions except for road transport in ventilated tunnels, air transport at 200 m above ground or higher, water transport, and biogenic particles. The effect of these noninventoried emissions, albeit negligible [< 1.0 μg/m^3^ (SAEFL 2003)], was implicitly included in the background concentration, which primarily accounted for imported primary and secondary particles. The primary background PM_10_ included Sahara desert sand events, biogenic materials, mineral dust and sea salt aerosols, noninventoried Swiss emissions, and other anthropogenic particles from abroad. The background primary and secondary concentrations were not dispersed with the dispersion model. They were first determined from the difference between the modeled results from Swiss emissions only and the measurements at Basel, Bern, Payerne, and Zurich, with the differences representing the sum of the background emissions and the model errors. The total background concentration for any given location was then computed using an elevation and region dependent empirical nonlinear function [Supplemental Material, Equation 1 (online at http://www.ehponline.org/docs/2007/10177/suppl.pdf)].

For NO_x_, the background concentration covered the natural sources, the far-reaching impact of Swiss emission sources, the total impact of any NO_x_ sources not covered by the emission inventory, and the regional anthropogenic background. Thus, the background concentrations included both long-range transported pollutants from abroad (including possibly traffic exhaust) and within Switzerland, whereas the traffic-specific concentrations covered exclusively emissions from urban sources.

We modeled the PM_2.5_ concentrations separately based on the PM_10_ emissions and source-specific factors describing the share of the fine fraction on the total PM_10_. Separate transfer functions for the fine fractions with different particle deposition velocity were used. For the background PM_2.5_ concentration, we estimated a weighted mean of 93% of the background PM_10_. We calculated concentrations of NO_2_ from the total NO_x_ estimates using the plume volume molar ratio method (Hanrahan 1999a) with the conversion coefficients derived from actual NO_2_/ NO_x_ observations across Switzerland.

### Analysis

We evaluated the dispersion model results for PM_10_, NO_x_, and NO_2_ against measurements in 1990 and 2000. The PM_2.5_ predictions were not evaluated because few measurements were available. The modeled city means of PM_10_ and NO_2_ were examined against the measured city means. We examined the within-city modeling error by calculating the Pearson’s and Spearman’s correlation coefficients (*r*_P_ and *r*_S_) between the deviations of modeled and measured values from their city means from cities with at least two monitoring sites. For NO_2_, we evaluated modeling error against the central-site and home outdoor measurements, respectively. Model predictions for traffic-specific PM_10_, PM_2.5_, NO_x_, and NO_2_ were evaluated against NO_x_ measurements. Because of the significant correlations among sources, we used principal component analysis (PCA) to identify principal components (or the source groups) that explained most of the variability in the predicted outdoor PM_10_. Source-specific NO_2_ was not examined because the dispersion model only computed total NO_2_ based on total NO_x_.

### Estimating annual means

To estimate the annual PM_10_ averages between 1990 and 2000 for individual residences, we used the modeled values at individual residences in 1990 and 2000, and the 1990–2000 historical trends of central-site measurements to develop an algorithm to interpolate dispersion modeled values. To assess historical trends, we defined areas with comparable sources or climatic characteristics, including the catchment areas of the eight study centers, Zurich, and Bern; each area is represented by at least one monitoring station. Residences outside these areas were grouped into four “other” areas based on similarities in meteorology and pollution sources. PM_10_ in any year *t* at station *i*, PM*^i^*_10_(*t*), was expressed as:


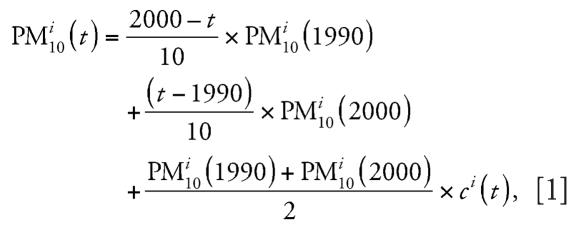


where the interpolation term *c^i^*(*t*) represents relative deviations from the simple linear interpolation:


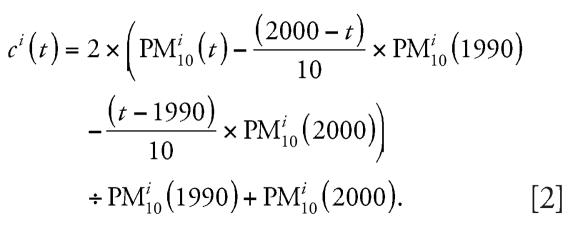


*c^i^*(*t*) vanishes if and only if the respective annual means lie on the straight line connecting the 1990 and the 2000 annual means. To obtain more robust estimates of *c^i^*(*t*), local interpolation terms were shrunk using an empirical Bayes methodology. For this purpose, all available PM_10_ and TSP annual means from all stations with complete data between 1990 and 2000 were used (*n* = 18). Correlation analyses suggested that the interpolation term *c^i^*(*t*) exhibited a similar longitudinal pattern for most of the 16 stations north of the Alps with complete PM_10_ data since 1990. For these sites, we averaged all local interpolation terms to obtain an annual mean interpolation term, *c*(*t*) (*t* = 1990,…,2000). For shrinkage, we decomposed the variance of the local interpolation term *c^i^*(*t*) of a given year *t* into an across-areas variance ρ*t*^2^ and a within-areas variance σ*t*^2^ and estimated σ*t*^2^ using the data from the four areas (Aarau, Berne, Geneva, and Zurich) with at least two stations. We then shrunk local interpolation term *c^i^*(*t*) toward the average interpolation term *c*(*t*) using the formula:


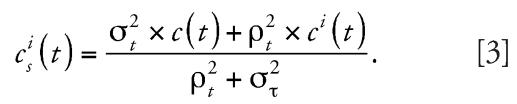


The assignment of *c^i^_s_*(*t*) is based on the area assignment of individual residences.

## Results

### Summary of measurements

Table 1 summarizes available annual averages of PM_10_, PM_2.5_, NO_x_, NO_2_, and BS measurements at the eight SAPALDIA areas in 1990 and 2000. A significant decrease in PM_10_, NO_x_, and NO_2_ levels between 1990 and 2000 was observed in all areas, with a larger reduction in more polluted areas. PM_2.5_ was not monitored until 1998. Lugano had the highest levels for all measurements in 2000.

### Evaluation of dispersion model predictions

**Figure 1:**
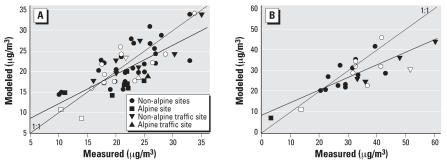
Measured versus modeled annual PM_10_ concentrations for all Swiss sites including those in the SAPALDIA areas (in white) in (*A*) 2000 [SAPALDIA areas: *y* = 1.9 + 0.87*x* (*R*^2^ = 0.68, *n* = 15); all sites: *y* = 5.2 + 0.72*x* (*R*^2^ = 0.55, *n* = 57] and (*B*) 1990 [SAPALDIA areas: *y* = 8.6 + 0.6*x* (*R*^2^ = 0.45, *n* = 8); all sites: *y* = 8.6 + 0.61*x* (*R*^2^ = 0.63, *n* = 25)]. Traffic sites are within 20 m of a major road.

For PM_10_ in 2000, the dispersion model predicted at least 55% of the variability in the measurements (Figure 1A). The dispersion model generally underestimated PM_10_ in the alpine region, due partially to the oversimplified alpine meteorology. For PM_10_ in 1990, the model underestimated most measurements, due potentially to the less accurate emission inventory data. In 2000, the model performed equally for background and traffic sites (e.g., sites located within 20 m of a major road and shown as up or down triangles) (Figure 1A, Table 2).

**Figure 2:**
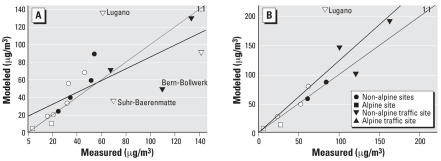
Measured vs. modeled annual NO_x_ concentrations for all Swiss sites including those in the SAPALDIA areas (in white) in (*A*) 2000 [SAPALDIA: *y* = 17.1 + 0.68*x* (*R*^2^ = 0.43, *n* = 10); all sites: *y* = 18.6 + 0.67*x* (*R*^2^ = 0.48, *n* = 17)] and (*B*) 1990 [SAPALDIA areas: *y* = −39 + 2.4*x* (*R*^2^ = 0.79, *n* = 6); all sites: *y* = −0.1 + 1.2*x* (*R*^2^ = 0.66, *n* = 11)]. Traffic sites are shown as triangles.

**Figure 3:**
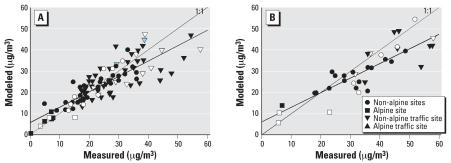
Measured vs. modeled annual NO_2_ concentrations for all Swiss sites including those in the SAPALDIA areas (in white) in (*A*) 2000 [SAPALDIA areas: *y* = 3.3 + 0.81*x* (*R*^2^ = 0.79, *n* = 24); all sites: *y* = 5.8 + 0.73*x* (*R*^2^ = 0.72, *n* = 103)] and (*B*) 1990 [SAPALDIA areas: *y* = 1.8 + 0.78*x* (*R*^2^ = 0.80, *n* = 12); all sites: *y* = 6.0 + 0.69*x* (*R*^2^ = 0.75, *n* = 38)]. Two blue triangles are airport sites.

The NO_x_ model generally overestimated measured NO_x_ and did not predict well at some traffic sites (Figure 2, Table 2). For non-traffic sites, the *R*^2^ was 0.88 (*n* = 11) and 0.91 (*n* = 7) in 1990 and 2000, respectively. Although traffic sites were distinguishable by elevated NO_x_ measurements, they were indistinguishable in the modeled values in 2000 (Figure 2A). The outliers marked in Figure 2 were partially attributed to the approximately 100-m inaccuracy in the geographic information system (GIS) codes against the actual distance from the closest major street. For NO_2_, the model predicted well the measured values with an *R*^2^ of ≥0.72 (Figure 3). NO_2_ measurements at traffic sites were not necessarily higher than those at background sites. The dispersion model on average underestimated the NO_2_ measurements at the traffic sites by 3.1 and 7.7 μg/m^3^ in 2000 and 1999, respectively, and less so for background sites (Table 2).

The predicted and measured city means of PM_10_ were comparable, with a Spearman correlation coefficient of 0.71 (*r*_P_ = 0.71, *n* = 57) [Supplemental Figure 1A (online at http://www.ehponline.org/docs/2007/10177/suppl.pdf)]. The modeled within-city residuals were comparable to and correlated with the measured values (*r*_S_ = 0.60; *r*_P_ = 0.79), indicating that the dispersion model was able to distinguish locations with higher concentrations from those with lower concentrations within a city. For NO_2_, the predicted city means correlated with those from the central-site measurements (*n* = 103, up to four sites per city) with an *r*_S_ of 0.88 (*r*_P_ = 0.85), larger than that for the within-city residuals (*r*_S_ = 0.44, *r*_P_ = 0.80). Comparing the modeled values with those from the home outdoor NO_2_ measurements (*n* = 335, up to 54 sites per city), the *r*_S_ was 0.87 (*r*_P_ = 0.91) for city means and 0.52 (*r*_P_ = 0.59) for within-city residuals. These results distinguished the differences in the modeling error between PM_10_ and NO_2_ predictions. The PM_10_ predictions demonstrated consistency with the measured values across- and within-cities. Although the NO_2_ predictions were able to match the measured city means, the predicted within-city residuals were less consistent with those measured.

### Evaluation of predictions of traffic-specific pollutants

BS correlated extremely well with the NO_x_ measurements with an *R*^2^ of 0.99 [Supplemental Figure 2A (online at http://www.ehponline.org/docs/2007/10177/suppl.pdf)], higher than those for NO_2_, PM_10_, and PM_2.5_ (Supplemental Figure 2B–D). This near-perfect correlation between BS and NO_x_ indicated that NO_x_ and BS shared a common source. Because the number of available BS measurements was limited, we thus used NO_x_ as the reference to evaluate traffic-related pollutant predictions.

**Figure 4:**
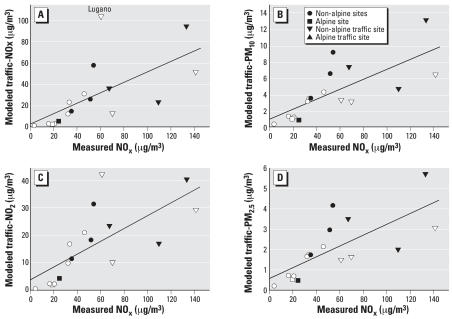
Relationship in 2000 between measured NO_x_ and modeled traffic-related (*A*) NO_x_ [background sites: *y* = −14 + 0.98*x* (*R*^2^ = 0.77, *n* = 11); traffic sites: *y* = 41 + 0.13*x* (*R*^2^ = 0.01, *n* = 6); all sites: *y* = 2 + 0.50*x* (*R*^2^ = 0.41, *n* = 17)]; (*B*) PM_10_ [background: *y* = −2 + 0.16*x* (*R* ^2^ = 0.84, *n* = 11); traffic sites: *y* = 0.4 + 0.06*x* (*R*^2^ = 0.35, *n* = 6); all sites: *y* = 1 + 0.06*x* (*R*^2^ = 0.54, *n* = 17)]; (*C*) NO_2_ [background: *y* = −7 + 0.59*x* (*R* ^2^ = 0.85, *n* = 11); traffic sites: *y* = 20 + 0.08*x* (*R*^2^ = 0.04, *n* = 6); all sites: *y* = 4 + 0.24*x* (*R*^2^ = 0.50, *n* = 17)]; and (*D*) PM_2.5_ [background: *y* = −0.7 + 0.07*x* (*R*^2^ = 0.86, *n* = 11); traffic sites: *y* = 0.4 + 0.03*x* (*R*^2^ = 0.34, *n* = 6); all sites: *y* = 0.6 + 0.03*x* (*R*^2^ = 0.53, *n* = 17)]. SAPALDIA sites are in white.

Figure 4 shows the predicted versus measured traffic exhaust pollutants. Measured NO_x_ levels clearly distinguished traffic from background sites, but the modeled values could not. Similar prediction profiles were observed for NO_x_ and NO_2_ and for PM_10_ and PM_2.5_ because the dispersion model estimated NO_2_ and PM_2.5_ from NO_x_ and PM_10_, respectively. The model clearly predicted better at the background sites than at the traffic sites.

### Time trend of emissions

The three largest local PM_10_ emission sources included industry, traffic, and household [Supplemental Table 2 (online at http://www.ehponline.org/docs/2007/10177/suppl.pdf)]. Emissions from all sources decreased between 1990 and 2000. For PM_10_, the largest reduction (43%) occurred in the industrial source. For NO_x_, traffic was the largest source, with emissions approximately three times higher than those of other two large sources, household and industry [Supplemental Table 3 (online at http://www.ehponline.org/docs/2007/10177/suppl.pdf)]. The largest reduction of 43% between 1990 and 2000 occurred in traffic emissions.

**Figure 5:**
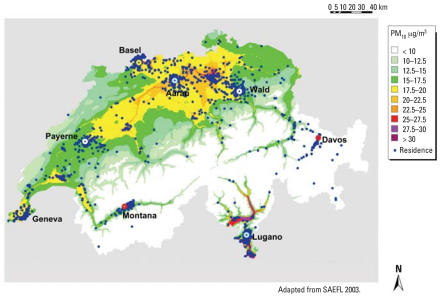
Dispersion modeling predictions for PM_10_ concentrations outside residences of the SAPALDIA 2 cohort.

The dispersion results produced concentrations of PM_10_, PM_2.5_, NO_x_, and NO_2_ for all of Switzerland and were spatially interpreted to coordinates of individual addresses (e.g., Figure 5). Table 3 shows the average individual source-specific concentrations of PM_10_ in each area for those who did not move between SAPALDIA 1 and 2. Home outdoor concentrations of PM_10_ were the highest in urban areas and lowest in the Alpine areas. The major emission contributors of PM_10_ are not necessarily the major contributors of the predicted concentrations due to the interplay among the dispersion process, long-range transport, atmospheric chemical processes, and the home locations. Background PM_10_ accounted for an average of 54% of the PM_10_ mass concentrations, followed by secondary PM_10_ except for Basel and Geneva. The spatial variation of total PM_10_ within each area, expressed as the coefficient of variation (CV), was small (range, 2–11%) (Table 3). Background and secondary PM_10_ were generally homogeneous within areas except for Montana and Lugano (Table 3) due to the elevation-dependent background influence. Although the contribution of traffic-originated PM_10_ from urban sources accounted for < 20% of the total PM_10_ mass concentrations, it was highly variable within areas (range of CV, 16–35%). Similar results were found for the predicted 1990 PM_10_ (results not shown).

The highest and most variable NO_x_ predictions were found in larger cities (Table 4). NO_x_ from traffic emissions accounted for most of the NO_x_ mass concentration in all four urban areas (Basel, Geneva, Aarau, and Lugano) and had the highest CVs within all areas (range, 30–86%) (Table 4). Background NO_x_ was the next largest contributor to total NO_x_.

**Figure 6:**
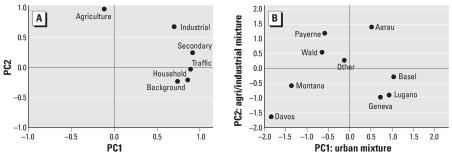
(*A*) Two principal components of sources which are presented by their loadings on these two components. (*B*) Source characteristics of the SAPALDIA areas given by the two component scores. “Other” includes subjects who have moved out of the SAPALDIA areas.

The PCA analysis identified two principal components (PCs) accounting for 82% of the variability in the predicted PM_10_ concentrations. The most important PC was the “urban mixture” including secondary, traffic, household, background, and industrial PM_10_, accounting for 56% of the variability (Figure 6A). The second PC was a mixture of agricultural and industrial PM_10_ and to a lesser extent secondary PM_10_, which accounted for 26% of the variability. The scores of these two PCs at individual residences were then averaged over each area (Figure 6B). Residences in Basel, Geneva, and Lugano scored positively (above the average) on the “urban mixture” and negatively on the “agricultural/industrial mixture.” Two rural areas, Payerne and Wald, scored positively on “agricultural/industrial mixture” and negatively on the “urban mixture.” The alpine areas, Davos and Montana, scored negatively on both mixtures, whereas Aarau scored positively on both mixtures. Those who moved out of our study areas (“other”) scored near the average (zero) on both mixtures.

### Cumulative versus differences in exposure

We evaluated the interpolated yearly PM_10_ predictions between 1990 and 2000 against PM_10_ measurements from Basel, Lugano, and Payerne, where measurements were available throughout the 11 years. The predicted yearly PM_10_ agreed well with the measured annual means with an *R*^2^ value between 0.74 and 0.80. Based on model predictions, we reconstructed annual exposures for every subject and calculated the cumulative exposure and the changes in exposure between 1990 and 2000. There was a clear inverse relationship between the cumulative exposure and the exposure reduction between 1990 and 2000 (*r* = −0.99) [Supplemental Figure 3 (online at http://www.ehponline.org/docs/2007/10177/suppl.pdf)]. Although the variability in the cumulative exposure and exposure reduction is large within larger cities, general linear model results for nonmovers indicated that the between-cities variance accounted for 88% and 92% of the variability in the estimated individual cumulative and changes in exposures to PM_10_ between 1990 and 2000, respectively.

## Discussion

We assessed the performance of the dispersion model for total PM_10_, NO_x_, and NO_2_ based on the agreement between the total predictions and measurements, between traffic-specific predictions and NO_x_ measurements (a traffic marker), and between the predicted and measured variations within and between cities. PM_10_ concentrations at background locations were appropriately predicted by the model. PM_10_ and NO_2_ concentrations at traffic sites, especially those with heavy traffic, were underestimated. NO_x_, a better traffic marker than NO_2_, was more difficult to predict locally due to its reactivity and thus the large spatial gradient within an area (Hanrahan 1999a, 1999b). At the current spatial resolution (200 × 200 m), the model could not accurately predict NO_2_ and NO_x_ at locations that are strongly affected by local conditions. On further examination, we discovered that the imprecision of the GIS data, such as the address codes and thus the distance to major roads, could be as large as 100 m, depending on the geocoding algorithm. This would result in an imprecision of our spatially interpreted dispersion model predictions in neighborhoods with a high spatial variability in pollutant concentrations.

For PM_10_, the modeled and measured city means and within-city residuals were comparable due to the small spatial variability of PM_10_. Our results indicated that the PM_10_ dispersion model was able to distinguish locations with higher concentrations from those with lower concentrations within and between cities. In contrast, the within-city residuals for NO_2_ were less consistent with the measured values, stressing the need for further model refinement to take into account local geographic and emission characteristics. Although our analysis of within-city modeling error for PM_10_ was performed with a limited numberof monitoring sites within cities, our results agreed with those of Cyrys et al. (2005), who reported small and very agreeable CVs for the measured PM_2.5_ (13.2%) at 40 sites and the dispersion modeled TSP (12.9%).

Although PM emissions from industry and traffic were the largest among all domestic sources, the dispersion model predictions indicated that background particles accounted for the largest share (mean = 54%) of the ambient PM mass concentration. This large background contribution might have been inflated by the implicit inclusion of the model error. However, the magnitude of the background contribution agreed with those (50–65% at European urban background-sites) reported by Querol et al. (2004), who used a source apportionment method. Model predictions demonstrated a clear within-area spatial variation for traffic-specific PM_10_ and for total and traffic-specific NO_x_ (Tables 3 and 4). As in most cohort studies, health effect estimates for SAPALDIA 1 subjects were based on the central-site measurements (Ackermann-Liebrich et al. 1997). Without taking into account these differences in individual exposures, misclassification of exposures among subjects might weaken the association with the health effects. The bias in the health effect estimates also may vary by source-specific exposure as indicated by their different variances. Health effect assessment using these improved source-specific and historical individual exposure estimates should shed further insights to the effects of long-term air pollution exposure.

With these source-specific exposure estimates, the SAPALDIA areas were clearly distinguished by the relative impacts of urban sources and a mixture of agriculture and industrial sources. Thus, PM constituents may differ by area. Although effects by area were demonstrated previously (Samet et al. 2000), no studies had the tools to examine health effects by source over a long period. We devised methods to reconstruct individual exposure history and observed a strong negative correlation between cumulative exposure and exposure reduction [Supplemental Figure 3 (online at http://www.ehponline.org/docs/2007/10177/suppl.pdf)]. We believe that this reflects a situation typical in many areas of the world where air pollution abatement policies were implemented during the last decades with a focus on more polluted areas (i.e., high cumulative exposure). As a result, changes (or improvements) are larger in these areas. This paradox needs to be considered in long-term air pollution studies because it may seriously influence the ability to observe health effects and the interpretation of findings. Depending on the health outcomes, the more recent changes in air quality may be more important than the long-term cumulative exposure, or vice versa.

The strengths of this study include the evaluation of the model predictions based on actual measurements over 2 separate years at up to 103 sites of various geographic characteristics. We are not aware of any studies that provided such comprehensive evaluation for assessing dispersion model predictions due partially to the difficulties of data collection. Bellander et al. (2001) modeled NO_2_ from road traffic and SO_2_ from house heating, with no source-specific measurements to evaluate their predictions. Wu et al. (2005) predicted time-location weighted exposure estimates from transport without measurements to evaluate their predictions.

No epidemiologic studies thus far have used the dispersion model approach to estimate and examine PM exposure from various sources. Few studies have focused on dispersion model predictions from traffic or home heating sources (Gauderman et al. 2005; Künzli et al. 2000; Pierse et al. 2006), with limited or no validation of the model predictions. Other studies have used the receptor modeling approach to apportion sources of central-site PM_2.5_ to represent the average population exposure (Laden et al. 2000; Mar et al. 2000; Tsai et al. 2000). Such an approach is subject to the availability of the speciated data at few receptor sites, and misses the substantial spatial variation of traffic-specific exposure within a city. The dispersion modeling approach described in this article uses emissions from different source categories and local meteorologic parameters to predict source-specific exposures outside residences and provides spatially resolved exposure for examining source-specific health effects.

One disadvantage of the dispersion model is its dependence on the availability and quality of the emission inventory data, which continue to become more available as required by the regulatory agencies. As the GIS evolves, we expect the accuracy and precision of GIS and traffic emission data to improve over time. One weakness of our dispersion model is the conversion of NO_2_ from total NO_x_ based on a general first-level conversion equation for all Swiss locations. The NO_x_–NO_2_ conversion depends on temperature, solar radiation, zenith angle, background ozone, and sources (Hanrahan 1999a,b). Although the spatial resolution of our current model was one of the finest, as compared with the 5 × 5-km resolution described by Wu et al. (2005) and Hoffmann et al. (2006), and between 100 × 100 m to 2 × 2 km by Bellander et al. (2001), it still did not provide sufficient resolution to clearly distinguish traffic exposures. Improvements of the dispersion model could be achieved with better traffic emission data, location and source-specific NO_x_–NO_2_ conversion factors, a higher spatial resolution, and/or the inclusion of a traffic emission submodule (Pierse et al. 2006).

## Conclusions

In this article we provide a comprehensive evaluation of the dispersion modeling estimates for all and for traffic-specific sources. For PM_10_, the dispersion model is suitable for estimating and comparing individual exposures between and within cities. Individual estimates for NO_2_ within a city, however, need further refinement. As better emission and high-resolution GIS data become more available, the dispersion modeling approach employing both physical and stochastic processes should provide a great tool for individual source-specific exposure estimates in air pollution health assessment studies.

## Figures and Tables

**Table 1:** Annual averages (± SD) of air pollution measurements in the eight SAPALDIA study areas.

		1990^a^			2000^a^				
Area	Altitude (m)	PM_10_ (μg/m3)	NO_x_ (ppb)	NO_2_ (μg/m3)	PM_10_ (μg/m3)	PM_2.5_ (μg/m3)	NO_x_ (ppb)	NO_2_ (μg/m3)	BS^b^ (m^−1^)
Basel	320	33.2 ± 18.7	39.3 ± 28.5	40.5 ± 18.4	20.5 ± 11.5	15.8 ± 10.4	20.4 ± 15.1	24.8 ± 12.1	1.0 ± 0.5
Wald	640	[Table-fn tfn1-ehp0115-001638]—	[Table-fn tfn1-ehp0115-001638]—	[Table-fn tfn1-ehp0115-001638]—	15.7 ± 8.6	11.7 ± 7.0^c^	9.5 ± 6.9	3.6 ± 7.4	0.8 ± 0.3
Davos	1,637	13.8	16.4 ± 12.3	23.1 ± 13.1	14.0 ± 7.9	6.9 ± 3.8^c^	11.6 ± 11.9	5.1 ± 12.9	[Table-fn tfn1-ehp0115-001638]—
Lugano	281	41.7 ± 22.1	53.7 ± 38.7	51.9 ± 19.0	33.8 ± 23.6	24.9 ± 19.1	39.1 ± 25.9	38.9 ± 15.2	1.5 ± 0.6
Montana	1,350	[Table-fn tfn1-ehp0115-001638]—	[Table-fn tfn1-ehp0115-001638]—	[Table-fn tfn1-ehp0115-001638]—	10.4 ± 6.2^c^	8.1 ± 4.9^c^	[Table-fn tfn1-ehp0115-001638]—	[Table-fn tfn1-ehp0115-001638]—	0.5 ± 0.3
Payerne	490	29.0 ± 17.1	14.6 ± 9.0	18.3 ± 8.7	19.8 ± 12.3	14.7 ± 10.3	12.5 ± 8.9	15.9 ± 8.5	0.9 ± 0.4
Aarau	417	41.4	[Table-fn tfn1-ehp0115-001638]—	36.7	28.7 ± 15.3	21.6 ± 13.3	[Table-fn tfn1-ehp0115-001638]—	[Table-fn tfn1-ehp0115-001638]—	1.2 ± 0.5
Geneva	375	51.6	[Table-fn tfn1-ehp0115-001638]—	59.9 ± 21.1	21.7 ± 12.4	[Table-fn tfn1-ehp0115-001638]—	[Table-fn tfn1-ehp0115-001638]—	36.9 ± 12.8	[Table-fn tfn1-ehp0115-001638]—

—, no data. Mean values without SD in 1990 were annual means reported by SAEFL, where daily values were no longer available.

a Sample size (no.) ranges between 302 (83% of possible samples) and 366 for all reported annual averages except for those noted below.

b BS, presented as absorption coefficient, was taken 1 in 3 days. No. ranges between 84 and 122.

c No. ranges between 180 and 183 (every other day samples), except for Montana where *n* = 124.

**Table 2:** Ratios of the dispersion modeled to measured pollutant values and the differences (μg/m^3^).

		Ratio (modeled – measured)					Difference (modeled – measured)				
Pollutant (year)	Site	Mean	SD	Min	Max	No.	Mean	SD	Min	Max	No.
PM_10_ 2000	All	0.97	0.20	0.61	1.45	57	−1.0	3.9	−10.2	5.3	57
	Background	0.98	0.21	0.61	1.45	47	−1.0	4.0	−10.2	5.3	47
	Traffic	0.96	0.15	0.74	1.14	10	−1.3	3.8	−6.7	3.4	10
PM_10_ 1990	All	0.93	0.29	0.60	2.05	25	−4.4	7.2	−20.9	5.3	25
	Background	0.98	0.30	0.62	2.05	20	−2.1	5.7	−14.2	5.3	20
	Traffic	0.71	0.07	0.60	0.78	5	−13.5	5.2	−20.9	−7.3	5
NO_x_ 2000	All	1.10	0.47	0.45	2.24	17	0.9	31.1	−60.4	75.6	17
	Background	1.16	0.34	0.49	1.67	11	7.7	13.2	−10.0	35.0	11
	Traffic	0.98	0.66	0.45	2.24	6	−11.5	49.5	−60.4	75.6	6
NO_x_ 1990	All	1.17	0.53	0.46	2.57	11	17.3	42.0	−19.3	130.3	11
	Background	0.98	0.26	0.46	1.29	7	0.4	9.8	−15.0	17.7	7
	Traffic	1.51	0.75	0.84	2.57	4	46.7	62.3	−19.3	130.3	4
	Traffic^a^	1.16	0.31	0.84	1.46	3	18.9	34.1	−19.3	46.1	3
NO_2_ 2000	All	1.01	0.29	0.53	2.99	103	−1.1	6.0	−18.9	12.3	103
	Background	1.03	0.31	0.53	2.99	86	−0.7	6.2	−18.9	12.3	86
	Traffic	0.92	0.11	0.65	1.09	17	−3.1	4.1	−12.8	3.2	17
NO_2_ 1990	All	0.91	0.28	0.46	1.95	38	−5.3	7.5	−22.4	6.6	38
	Background	0.95	0.32	0.46	1.95	24	−3.8	6.0	−15.3	6.6	24
	Traffic	0.86	0.17	0.59	1.07	14	−7.7	9.4	−22.4	3.2	14

Abbreviations: Max, maximum; Min, minimum.

a Without Lugano.

**Table 3:** Source-specific PM_10_ (μg/m^3^), percentage of the total, and the CV within each source in 2000 among nonmovers.

Area	Traffic	Secondary	Background	Agricultural	Industrial	Household	Total	No.
Basel	4.4	4.0	12.9	0.9	2.3	1.1	25.5	515
% total	17	16	51	3	9	4	100	
CV (%)	32	1	3	6	10	26	7	
Wald	1.4	3.1	8.2	1.3	2.0	0.3	16.3	669
% total	9	19	50	8	12	2	100	
CV (%)	22	3	5	5	5	10	5	
Davos	0.8	1.3	4.9	0.4	0.8	0.2	8.3	294
% total	10	16	59	4	9	2	100	
CV (%)	29	3	3	16	10	23	4	
Lugano	3.0	3.9	22.2	0.5	2.1	0.7	32.4	559
% total	9	12	68	2	7	2	100	
CV (%)	35	9	10	9	18	29	11	
Montana	0.8	2.0	5.9	0.8	1.4	0.2	11.0	381
% total	7	18	53	7	13	2	100	
CV (%)	16	5	11	12	7	8	8	
Payerne	1.7	2.9	9.5	1.8	2.2	0.4	18.4	582
% total	9	16	52	10	12	2	100	
CV (%)	24	0	1	3	2	11	2	
Aarau	3.6	3.7	10.1	1.6	3.0	0.6	22.6	523
% total	16	16	45	7	13	3	100	
CV (%)	16	1	1	3	3	6	3	
Geneva	4.1	3.3	11.5	0.6	2.0	1.3	22.7	335
% total	18	14	50	3	9	6	100	
CV (%)	23	0	1	3	5	19	5	
Overall	2.5	3.1	11.1	1.1	2.1	0.6	20.5	3,858
% total	12	15	54	5	10	3	100	
CV (%)	61	25	46	47	27	64	35	

**Table 4:** Source-specific NO_x_ (μg/m^3^), percentage of the total, and the CV within each source in 2000 among nonmovers.

Area	Traffic	Background	Agricultural	Industrial	Household	Total	No.
Basel	31.8	18.9	1.6	6.1	7.6	65.9	515
% total	48	29	2	9	11	100	
CV (%)	42	36	3	20	13	25	
Wald	4.0	11.4	1.4	1.9	1.0	19.8	669
% total	20	58	7	10	5	100	
CV (%)	81	39	9	24	15	23	
Davos	2.2	3.0	1.0	1.7	1.1	8.9	294
% total	24	34	11	19	12	100	
CV (%)	86	42	10	31	17	26	
Lugano	51.8	17.6	2.6	5.3	5.0	82.3	559
% total	63	21	3	6	6	100	
CV (%)	55	33	9	23	17	37	
Montana	5.6	4.9	2.3	2.5	0.8	16.1	381
% total	35	31	14	16	5	100	
CV (%)	72	32	29	18	36	33	
Payerne	9.1	14.5	1.9	2.7	1.4	29.7	582
% total	31	49	6	9	5	100	
CV (%)	54	41	2	14	4	19	
Aarau	19.0	15.9	1.5	4.6	2.4	43.4	523
% total	44	37	4	11	5	100	
CV (%)	32	23	3	16	7	15	
Geneva	36.1	15.8	2.2	6.7	12.1	72.9	335
% total	50	22	3	9	17	100	
CV (%)	30	23	3	8	16	17	
Overall	20.3	13.5	1.8	3.9	3.6	43.1	3,858
% total	47	31	4	9	8	100	
CV (%)	105	104	37	49	32	68	
